# Effect of the Statistical Nature of Fiber Strength on the Predictability of Tensile Properties of Polymer Composites Reinforced with Bamboo Fibers: Comparison of Linear- and Power-Law Weibull Models

**DOI:** 10.3390/polym8010024

**Published:** 2016-01-21

**Authors:** Xue Li, Fang Wang

**Affiliations:** Faculty of Materials and Energy, Southwest University, Chongqing 400715, China; hcj504976580@swu.edu.cn

**Keywords:** natural fiber, Weibull statistics, failure behavior, strength variability, Monte-Carlo simulation

## Abstract

In fibrous composites, tensile strength of reinforcements exhibits a stochastic nature, and the mechanical properties of the composites are significantly influenced by such strength variability. The present study aims at providing a comparative investigation of the influence of the statistical variation in fiber strength on the tensile properties of unidirectional composites reinforced by bamboo fibers. Monte-Carlo simulations coupled with the linear- and power-law Weibull distributions are performed to conduct numerical predictions for damage evolution and strength variability of the composites, and the predicted mean strength and failure strain are compared with the experimental results. The Weibull parameters used are achieved through the Maximum Likelihood Estimation with multiple data sets of fiber lengths. Fiber strength statistics is found to have an effect on composite mechanical properties. The results further indicate that the use of the power-law model is relatively efficient for modeling purposes in comparison to the linear-law model, which could be attributed to fiber diameter variation.

## 1. Introduction

Fibers are the main load-bearing constituents in fiber-reinforced polymeric composites and some of them exhibit brittle fracture behavior. Owing to varying severities of defects that could have originated in the precursor material or have been induced during the fabrication process, the fibers are generally found to possess marked strength scatter [1,2]. Fiber breakages randomly occur during loading, which arise mainly from the variability in its fracture strength. The resulting local stress concentration takes place in the fibers and matrix regions in the vicinity of those broken sites, leading to the occurrence of various damage events prior to formation of a catastrophic crack. Therefore, the stochastic characteristic of the fiber strength is one of the most influential factors on the mechanical behavior of fibrous composites [3,4]. Modeling of the damage evolution and failure behavior in composites requires an extensive understanding of how fiber strength is scattered. Compared with normal and exponential distributions, Weibull statistics has been proposed on the assumption that the failure of material occurs when the weakest link fails [5,6] and is often used to characterize the statistical distribution of fiber strengths. The traditional Weibull description assumes that the number of flaws in a fiber is linear in the length and is a power-law in the stress [3]. That is, the scatter in fiber strength is described using a two-parameter Weibull distribution function [7], given as follows:

(1) $$P\left(\sigma_{f}\right)=1-\text{exp}\left\{-\left(\frac{L}{L_{0}}\right){\left(\frac{\sigma_{f}}{\sigma_{0}}\right)}^{\gamma }\right\}$$

where *P* is a failure probability of a fiber of length *L* up to a stress σ_f_. σ_0_ is the scale parameter that corresponds to a reference gauge length *L*_0_. γ denotes the shape parameter and is also an index of the variability of fiber strength: The strength distribution with a lower γ tends to perform larger scatter and *vice versa*. This equation is termed as the linear-law Weibull (LLW) model that directly links fiber strength to its length.

Although the above model is widely applied to many brittle fibers, an apparent discrepancy between the experimental data and model-derived predicted values appears when explaining the effect of fiber diameter variation on the fiber strength [8,9,10]. Indeed, the statistical character of brittle fibers is derived from those defects of random strengths distributed within the fiber in a random order [11]. Especially for fibers with considerable geometrical irregularities, it is evident that the distribution of flaws is inhomogeneous along the fiber length or between fibers, and then the presence of a distinct fluctuation of the density of flaws may lead to a large scatter of fiber strength [12,13]. Thus, the LLW model is incapable of accurately describing the fiber strength and the dependency of fiber strength on the material size. For example, natural fibers are sourced from the stem and leaves of plants. They are notably different from man-made fibers, which are characterized by within-fiber and between-fiber diameter variations. Significantly, inner geometrical structure and composition contribute to more scattered flaws within a fiber or between fibers, which result in a higher variation in fiber strength [14]. As a consequence, the validity of the LLW model has been widely questioned, and it is suggested that a modified Weibull form [15]

(2) $$P\left(\sigma_{f}\right)=1-\text{exp}\left\{-{\left(\frac{L}{L_{0}}\right)}^{\lambda }{\left(\frac{\sigma_{f}}{\sigma_{0}}\right)}^{\gamma }\right\}\left(\lambda \neq 1\right)$$

is preferable to Equation (1). Compared to the linear-law model, the above-modified equation introduces an exponential parameter *λ* that accounts for the strength variability, which is caused by the change in inherent flaw distribution not only along a fiber but also between fibers. Thus, the three-parameter model of Equation (2) is named as the power-law Weibull (PLW) model. It must be pointed out that although the exact physical meaning of *λ* was not clearly noted and even controversial [3,14], the physical origin of such a model is thought to be bound up with variation of strength characteristics caused by the random flaw distribution within the fiber [12,13].

Among the well-known natural fibers (jute, sisal, coir, *etc.*), bamboo fiber is recognized as one of the most competitive reinforcement fillers due to its outstanding inherent properties, such as low cost, high tensile modulus, and flexibility [16,17]. Also, the inexhaustible supply, natural abundance, and sustainability of this material make it an alternative to traditional synthetic fibers in structural composites. Over the last couple of years, the use of bamboo fiber as a reinforcing constituent has gained wide attention in the building and automotive industries [18]. In the case of fiber-reinforced composites, continuous fibers are used and usually occupy a considerable volume. Thus, the mechanical properties of composites are profoundly affected by the presence of variations in fiber strength [19,20], and also highly scattered in nature [21]. There is no denying that an accurate model for predicting the failure behavior of such composites relies on accurately characterizing fiber strength variability.

Despite the importance of the Weibull distribution in reliability analysis of brittle materials, systematic contrastive study between the two models has not been conducted for explicitly linking the fiber strength variation to the composite strength distribution [3,22,23]. Hence, the major focus of this work is to investigate the effect of the statistics of fiber strength on the tensile properties of bamboo fiber-reinforced composites. The accuracy of strength predictions using two- and three-parameter Weibull distribution is assessed in order to reveal which best matches the experiments. The Weibull parameters in the linear- and power-law model are calculated using the Maximum Likelihood Estimation (MLE) with multiple strength data at five different gauge lengths, including 20, 30, 40, 50 and 60 mm. Compared with experimental results, the validity of the Multiple Data Set (MDS) weak-link scaling predictions for fiber strength is examined as well.

## 2. Experimental Details

### 2.1. Materials

The fibers used in this study were sourced from Moso bamboo and were originally grown in Japan. They were delivered from Ban, Ltd. (Tokushima, Japan), which specializes in the production of bamboo fibers. All of the fibers were extracted out of the culms of bamboo plants of at least three years of age using a blasting extraction method. Like many plant-based fibers with geometrical irregularities, these fiber samples were found to exhibit a variation in diameter between fibers and within an individual fiber. However, our previous study revealed that the diameter variability along the fiber length was insignificant [17]. In addition, much work treated natural fibers as perfectly round and having a constant diameter for the purpose of analytical simplicity [17,24,25,26]. Herein, the fibers used in this study were assumed to be cylindrical in shape, and then the diameter of each individual fiber was determined from observations using an optical microscope as the average of apparent diameter measurements taken at five different locations along the gauge length. Afterwards, the bamboo fibers were employed to produce sets of samples with 20, 30, 40, 50, and 60 mm gauge lengths. It could be found from Table 1 that bamboo fibers exhibit between-fiber diameter variations at each tested length group. This scatter is known to be as a result of non-uniform geometrical structure due to growing conditions and moisture content [13,14].

**Table 1:** Variations in between-fiber diameters for each of five testing groups.

Gauge length (mm)	Number of available specimens, *N*	Average diameter (μm)
20	45	366.8 (20.5)
30	49	394.7 (22.4)
40	39	413.4 (26.2)
50	42	389.7 (23.0)
60	39	386.3 (28.8)

Values in parenthesis denote the standard deviations.

Prior to composite processing, the bamboo fibers were washed several times with distilled water to remove any dirt from the fiber surface. After that, they were dried at room temperature for 48 h, followed by drying in an oven at 80 °C for 2 h. The matrix used in this study was a commercial epoxy resin CYDF-175 supplied by Baling Petrochemical Corporation, Ltd. (Yueyang, China). The prepared mold was filled with an appropriate amount of the mixture of epoxy resin and bamboo fibers, and the corresponding volume fraction ratio was kept at 68:32. A vulcanization molding machine (XLB-0050, Jinrunqi, Qingdao, China) was used to preheat the samples for 10 min at 80 °C without pressure to allow permeation of the polymer through the fibers, and then the samples were kept at 100 °C and pressed for 5 h at a pressure of 15 MPa. The prepared composite plate was removed from the mold after curing at room temperature for 11 h and then at 80 °C at atmospheric pressure for 8 h. The straight-bar samples were rectangular coupons cut from this plate to the required dimensions of length 150 mm, width 20 mm, and thickness 2 mm by using a dedicated cutting machine with a diamond-coated cutting blade. In this work, configuration was limited to unidirectional, continuous bamboo fibers equal to the length of specimen; that is, 150 mm in the case of tensile testing. The selected laminate was a single lamina with a ply angle of 0°.

### 2.2. Tensile Tests

Fifty individual fibers were tested at each of the five groups, and each test was conducted on a WDW3050 universal testing machine (Kexin Testing Instrument Co., Ltd., Changchun, China) in accordance with ASTM D3379-75 [27]. The cross-head speed was 0.5 mm/min, and all of the static tests were carried out at a room temperature of 25 °C and average humidity of 50%, under atmospheric pressure. Tensile strength was calculated from the load–elongation data and fiber cross-sectional area. It needs to be noted that since samples that broke near the edge of the clamps had been excluded from the analysis, a final total of 214 fiber tests were used to measure fiber strength statistics, as shown as in Table 1.

All quasi-static tests were conducted in five composite samples tested on the above testing machine with a software package in accordance with ASTM D638-10 [28], which were under displacement control at a constant crosshead speed of 0.5 mm/min at room temperature in air. During the tensile test, the specimens were clamped at both ends in the chuck to guarantee the synchronization of the strain increasing in the fiber phase and matrix phase. They were held under a quasi-static monotonic loading until tensile fracture occurred, the time-to-failure being defined as the time at which the laminate could no longer support the externally applied load. Therefore, the tensile strength was taken as the maximum load divided by the cross sectional area of the specimen.

## 3. Theoretical Model

### 3.1. Weibull Distribution

In Equations (1) and (2), *P* is an unknown probability of failure, which can be estimated based on the following observations. If *n* samples are ranked in ascending order, σ_fi_ (*i* = 1 − *n*) is denoted as the *i*th strength value. Then, *P* is the expectation of σ_fi_ and is achieved by the statistical approximation technique [29].

For a constant tested length, rearranging and taking the double natural logarithms of both sides in Equation (1) or (2) can lead to a linear regression model in the form *Y* = γ*X* + *A*, where $Y=\text{ln}\left(\text{ln}\left(\frac{1}{1-P}\right)\right)$, $X=\text{ln}\left(\sigma_{\text{fi}}\right)$, and $A=-\gamma \;\text{ln}\left(\sigma_{0}\right)$. Hence, the scale and shape parameters can be easily obtained using the least square minimization, which will give a straight line with the gradient γ and the *Y*-intercept *A*.

It is well known that longer fibers should on average have lower fracture strength than shorter fibers, which can be explained by the fact that there is an increased probability of encountering a more severe flaw with the fiber length [1]. Further, several studies have demonstrated that the characteristic strength σ_0_ obtained at the reference gauge length *L*_0_ may be used to predict the strength at any given gauge length *L* [1,9]. The mean value of this random variable can be computed mathematically from Equations (1) and (2)

(3) $${\bar{\sigma }}_{f}=E\left(\sigma_{f}\right)=\sigma_{0}{\left(\frac{L}{L_{0}}\right)}^{-\frac{1}{\gamma }}\Gamma \left(1+\frac{1}{\gamma }\right)$$

(4) $${\bar{\sigma }}_{f}=E\left(\sigma_{f}\right)=\sigma_{0}{\left(\frac{L}{L_{0}}\right)}^{-\frac{\lambda }{\gamma }}\Gamma \left(1+\frac{1}{\gamma }\right)$$

where Γ represents the gamma function. The reference length *L*_0_ is generally normalized to 1 for mathematical convenience. The two equations are also considered the linear- and power-law Weibull model for strength scaling based on fiber length.

In addition, the variance of σ_f_ can be expressed as follows [30]

(5) $$\sigma_{f}^{2}=D\left(\sigma_{f}\right)=\sigma_{0}^{2}{\left(\frac{L}{L_{0}}\right)}^{-\frac{2}{\gamma }}\left\{\Gamma \left(1+\frac{2}{\gamma }\right)-{\left[\Gamma \left(1+\frac{1}{\gamma }\right)\right]}^{2}\right\}$$

(6) $$\sigma_{f}^{2}=D\left(\sigma_{f}\right)=\sigma_{0}^{2}{\left(\frac{L}{L_{0}}\right)}^{-\frac{2\lambda }{\gamma }}\left\{\Gamma \left(1+\frac{2}{\gamma }\right)-{\left[\Gamma \left(1+\frac{1}{\gamma }\right)\right]}^{2}\right\}$$

### 3.2. Characterization of Stress Profiles

Shear-lag analysis is one of the most popular models for characterizing stress profiles and simulating failure behavior in fiber-reinforced composites [31]. The longitudinal stress of unidirectional composite, σ_comp_, is given by

(7) $$\sigma_{\text{comp}}=V_{f}{\hat{\sigma }}_{f}+\left(1-V_{f}\right){\hat{\sigma }}_{m}$$

where *V*_f_ is the fiber volume fraction. ${\hat{\sigma }}_{f}$ and ${\hat{\sigma }}_{m}$ are defined as average fiber and matrix stress, respectively, as follows

(8) $${\hat{\sigma }}_{f}=\frac{1}{2N_{f}L_{T}}\sum_{k=1}^{N_{f}}\left[\int_{-L_{T}/l_{c}}^{L_{T}/l_{c}}\sigma_{f}(k,\xi )d\xi \right]$$

(9) $${\hat{\sigma }}_{m}=\frac{1}{2N_{m}L_{T}}\sum_{k=1}^{N_{m}}\left[\int_{-L_{T}/l_{c}}^{L_{T}/l_{c}}\sigma_{m}(k,\xi )d\xi \right]$$

Where σ_f_ and σ_m_ represent the axial stresses in fiber and matrix, respectively. *N*_f_ and *N*_m_ stand for the number of fiber and matrix in the composite specimen, respectively. 2*L*_T_ refers to the total length of the composite.

It is assumed that *N* breaks have appeared in fibers and each fiber break is followed by the interface splitting of length 2*L*_n_ (*n* = 1,2,…,*N*). Simultaneously, *M* breaks cause the associated matrix tensile cracking. In our simulations, the interface surrounding each break serves to transfer the lost fiber load to neighboring fibers and matrix through shear deformation. Under the framework of shear-lag arguments and influence superimposition technique [32], the axial stresses in the fiber and matrix regions and the interfacial shear stress at any position (*k*,ξ) are presented as follows

(10) $$\sigma^{f}\left(k,\xi \right)={\bar{\sigma }}_{f}+\sum_{j=1}^{N}\int_{\xi_{j}-L_{j}/l_{c}}^{\xi_{j}+L_{j}/l_{c}}w_{j}^{f}\left(\xi^{\prime }\right)p_{k-k_{j}}^{f}\left(\xi -\xi^{\prime }\right)d\xi^{\prime }+\sum_{i=1}^{M}w_{i}^{m}q_{k-k_{i}}^{f}\left(\xi -\xi_{i}\right)$$

(11) $$\sigma^{m}\left(k,\xi \right)={\bar{\sigma }}_{m}+\sum_{j=1}^{N}\int_{\xi_{j}-L_{j}/l_{c}}^{\xi_{j}+L_{j}/l_{c}}w_{j}^{f}\left(\xi^{\prime }\right)p_{k-k_{j}}^{m}\left(\xi -\xi^{\prime }\right)d\xi^{\prime }+\sum_{i=1}^{M}w_{i}^{m}q_{k-k_{i}}^{m}\left(\xi -\xi_{i}\right)$$

(12) $$\tau \left(k,\xi \right)=\sum_{j=1}^{N}\int_{\xi_{j}-L_{j}/l_{c}}^{\xi_{j}+L_{j}/l_{c}}w_{j}^{f}\left(\xi^{\prime }\right)s_{k-k_{j}}\left(\xi -\xi^{\prime }\right)d\xi^{\prime }+\sum_{i=1}^{M}w_{i}^{m}t_{k-k_{i}}\left(\xi -\xi_{i}\right)$$

where ${\bar{\sigma }}_{f}$ and ${\bar{\sigma }}_{m}$ are the fiber and matrix stress without any damage. The quantities of $p_{k-k_{j}}^{f}\left(\xi -\xi_{j}\right)$, $p_{k-k_{j}}^{m}\left(\xi -\xi_{j}\right)$, and $s_{k-k_{j}}\left(\xi -\xi_{j}\right)$ are called as the influence functions, which represent fiber axial stress, matrix axial stress, and interface shear stress, respectively, at position (*k*, ξ) due to a unit opening displacement exerted upon the fiber break at position (*k_j_*, ξ_*j*_). Accordingly, $q_{k-k_{i}}^{f}\left(\xi -\xi_{i}\right)$, $q_{k-k_{i}}^{m}\left(\xi -\xi_{i}\right)$, and $t_{k-k_{i}}\left(\xi -\xi_{i}\right)$ are the fiber axial stress, matrix axial stress, and interface shear stress, respectively, at position (*k*, ξ) due to a unit opening displacement exerted upon the matrix break (*k_i_*, ξ_*i*_). The undetermined weight functions $w_{j}^{f}$ (*j* = 1, 2,…, *N*), $w_{i}^{m}$ (*i* = 1, 2,…, *M*), and the splitting length 2*L_j_* (*j* = 1, 2,…, *N*) can be obtained by the boundary conditions. A detailed description of analytical solutions is provided in the literature [33,34].

It must be pointed out that the key concept of the solution is that the stress at any position is a weighted total of all loads transmitted by the individual damage, which takes into account the interaction of multiple damages. Interestingly, the computing time mainly involves solving for the weighting factors and just depends on the amount of damaged elements rather than composite size.

### 3.3. Monte-Carlo Simulation

Consider a two-dimensional unidirectional composite, in which fibers and matrix are arranged alternately. The composite mentioned is partitioned into *N*_L_ fiber elements along the direction of fiber length. If the total length of composite specimen is modeled, 2*L*_T_, then segment length Δ*x* is equal to 2*L*_T_/*N*_L_. When a failure probability *P* is given, the strength of fiber segments with length Δ*x* can be derived from the inversion of Equations (1) and (2), respectively,

(13) $$\sigma_{f}=\sigma_{0}{\left\{\left(\frac{L_{0}}{\Delta x}\right)\text{ln}\left(\frac{1}{1-P}\right)\right\}}^{\frac{1}{\gamma }}$$

(14) $$\sigma_{f}=\sigma_{0}{\left\{{\left(\frac{L_{0}}{\Delta x}\right)}^{\lambda }\text{ln}\left(\frac{1}{1-P}\right)\right\}}^{\frac{1}{\gamma }}$$

Thus, the random fracture strength σ_f_ can be obtained by generating uniform random number *P* in the the range of [0,1].

Monte-Carlo simulations have been used to compare these two classical Weibull methods used in the prediction of tensile strength for composites. The modeling is implemented by an incremental displacement-controlled loading, and the failure stress of the matrix and interface are considered to be definite. Prior to each simulation, all constituent elements are considered to keep intact. The simulation procedure can be outlined below:

(a) In view of the facts of the use of a MDS weak-link scaling method and the strength variability caused by the random distribution of flaw density due to inner geometrical structure and composition, it is reasonable to assume that each fiber element is independent and its strength identically follows a Weibull distribution when the discretization length is sufficiently small [34]. Then, assign a tensile strength to each fiber element according to LLW and PLW expressions.
(b) For a given applied load, the axial stress acting on the segments of the fiber and the matrix and the shear stress on the interface can be availably calculated by classical mechanical approach. Due to non-uniform fiber strength distribution, new fiber breakage happens in any position once the fiber stress is equal to corresponding strength.
(c) After the first fiber break takes place at early loading stage, the load carried by the broken fiber is redistributed, and it induces stress concentration close to the broken site. Thus, matrix transverse cracking will occur when the matrix stress reaches its tensile strength. Similarly, for interface, the interfacial splitting happens when the shear stress exceeds the shear strength on the segment of the interface. If the interface is split, a constant frictional stress is considered to act along the interface in the splitting zone, and then the interfacial shear stress will reduce to this friction stress [35]. Once any damage arises, stress distribution in the composite is recalculated and this step will be repeated until occurrence of new damage is terminated under the present loading level [34]. Afterwards, we calculate the composite stresses and go to step (d).
(d) Increase a new loading level and repeat steps (b) and (c). With a continuous accumulation of fiber breaks and the associated local damage events, the composite specimen will no longer be able to bear the externally applied load. Composite failure will occur when the tensile load is reduced to 85% of the maximum stress.

## 4. Results and Discussion

### 4.1. Analysis of Fiber Strength Distribution

It is well known that the tensile strength for any fiber length can be obtained from a single point estimate at a chosen gauge length with weak-link scaling. However, Virk *et al.* [26] recommended the use of Multiple Data Set (MDS) based on multiple gauge lengths when performing scaling predictions for fiber strength. In order to assess the accuracy of MDS estimates, the statistical parameters of the Weibull distribution are computed from the multiple data sets of fiber lengths for:

- 20 mm (to provide the shortest gauge length)
- 20 and 60 mm (to provide the extreme of gauge lengths)
- All gauge lengths (to provide all gauge lengths)

Table 2 gives the numerical values calculated by the MLE method, and then tensile strength at other gauge length is availably scaled on the basis of these parameters. It is observed that there is a distinct difference in Weibull statistics at 20 mm gauge length, which may be attributed to a consideration of variable fiber diameter in the power-law model.

**Table 2:** Weibull statistics for bamboo fiber strength.

Gauge length (mm)	Linear-law model		Power-law model		
	γ	σ_0_ (MPa)	γ	σ_0_ (MPa)	λ
20	5.2	11,00	4.8	692	0.19
20 and 60	5.3	1,093	4.2	1,115	0.85
All	5.1	1,109	4.2	1,087	0.85

The comparison of the predicted tensile strength at each gauge length with the experimental data is shown in Table 3. It is worth mentioning that the strength obtained is the average strength at each gauge length predicted with the LLW model by Equation (3) and the PLW model by Equation (4), respectively.

**Table 3:** Comparison of the predicted and experimental fiber strength at different gauge lengths.

Gauge length (mm)	Predicted strength (MPa)						Experimental data ** (MPa)
	*L* = 20 mm		*L* = 20, 60 mm		All lengths		
	LLW *	PLW *	LLW *	PLW *	LLW *	PLW *	
20	569 (4.2)	564 (3.3)	572 (4.8)	553 (1.4)	567 (3.8)	542 (0.8)	546 (145)
30	526 (4.2)	555 (9.9)	530 (4.9)	510 (1.0)	523 (3.6)	500 (1.0)	505 (130)
40	498 (4.2)	549 (14.8)	502 (5.0)	481 (0.6)	495 (3.5)	472 (1.4)	478 (122)
50	477 (9.4)	544 (2.7)	481 (10.4)	460 (5.5)	473 (8.6)	451 (3.4)	436 (115)
60	461 (9.9)	540 (28.9)	465 (11.0)	443 (5.8)	457 (9.0)	435 (3.7)	419 (112)

* Values in parenthesis denote difference error (%); ** Values in parenthesis stand for the standard deviation; LLW: Linear-Law Weibull model; PLW: Power-Law Weibull model.

From this table, there are two implications, as follows: First, with errors between the experimental data and predicted values, it is shown that the weak-link scaling based on a single point estimate at a chosen gauge length is an ineligible method, because the accuracy of the predictions is dependent upon the scaling point used. In fact, MDS estimates are derived from multiple points with various gauge lengths, and then this method can be generalized to perform scaling predictions for tensile strength at different fiber lengths [26]. Therefore, the Weibull parameters obtained from all the tested fiber lengths are used for the rest of the discussions. Second, the accuracy of the average strength values calculated by the PLW model is quantified and compared to the prediction with the LLW equivalent. The difference errors indicate that the three-parameter model seems to be more appropriate for the statistical description of tensile strength of bamboo fibers, because this modeling approach can yield a closer fit to the experimental data when performing the weak-link scaling for any gauge length.

To compare the predictability with the two models, the mean values and standard deviations for the fiber strength at different gauge lengths are utilized, as seen in Table 4. A general trend of decrease of tensile strength with increasing fiber length is observed, which could be derived from the fact that there is an increased probability of a flaw in a larger fiber. It also appears that unlike the PLW model, the bamboo fibers have a higher strength and exhibit less strength variability under the framework of the LLW model, which may be expected as a result of the non-uniform geometrical structure of natural fibers. The influence of fiber strength variability on composite mechanical behavior will be discussed in the subsequent analysis.

**Table 4:** Predicted fiber strength and its variability using Weibull distribution.

Gauge length (mm)	Tensile strength (MPa)			
	Linear-law model		Power-law model	
	Mean value using Equation (3)	Standard deviation using Equation (5)	Mean value using Equation (4)	Standard deviation using Equation (6)
20	567	127	542	144
30	523	118	500	133
40	495	111	472	126
50	473	106	451	120
60	457	103	435	116
150	382	86	362	96

### 4.2. Analysis of Composite Strength Distribution

Since this study is comparative in nature, two different Weibull models will be used in the analysis of composite strength distribution. The properties of the constituents are provided in Table 5. It is noted that the fiber segment length and the composite size need to be determined in advance. Our previous experiences show that the discretization length can be chosen as 1.0 mm for the purpose of computational accuracy and efficiency [34]. Accordingly, this value of Δ*x* will be used in our subsequent simulations.

**Table 5:** Mechanical properties of fiber and matrix.

Property	Fiber [29,36]	Matrix ^b^
Tensile strength, (MPa)	180–820 ^a^	65
Young’s modulus, (GPa)	12	2.7
Shear modulus, (GPa)	–	1.3
Shear strength, (MPa)	–	130

^a^ The two data represent the minimum and maximum values measured from different gauge lengths, respectively; ^b^ The value is provided by Baling Petrochemical Corporation, Ltd. (Yueyang, China).

**Figure 1:**
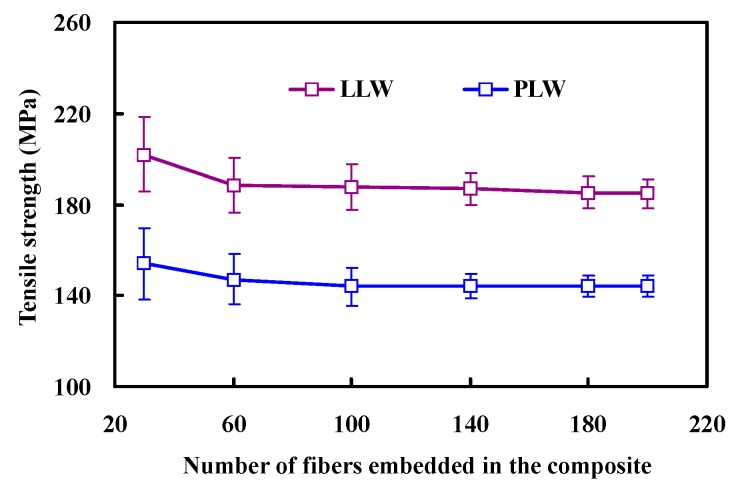
Dependence of tensile strength on the composite size.

To investigate the dependence of tensile strength on the composite size, a series of simulations based on the LLW and PLW model are performed to generate composite strength for the sample sizes of 30, 60, 100, 140, 180, and 200 fibers, as shown in Figure 1. For each data point, 200 simulations are carried out and the value given is the mean of those values.

Similarly, there is a size effect on the composite strength, where the average strength decreases with increasing number of fibers. The decreasing trend in tensile strength could be due to weak-link statistics, proving that a material with larger volume can be considered to have a larger number of links than a smaller one and therefore have an increasing probability of encountering a more severe flaw along the material [1]. Meanwhile, the results demonstrate that the dependence of strength on size disappears when the specimen is sufficiently large.

**Figure 2:**
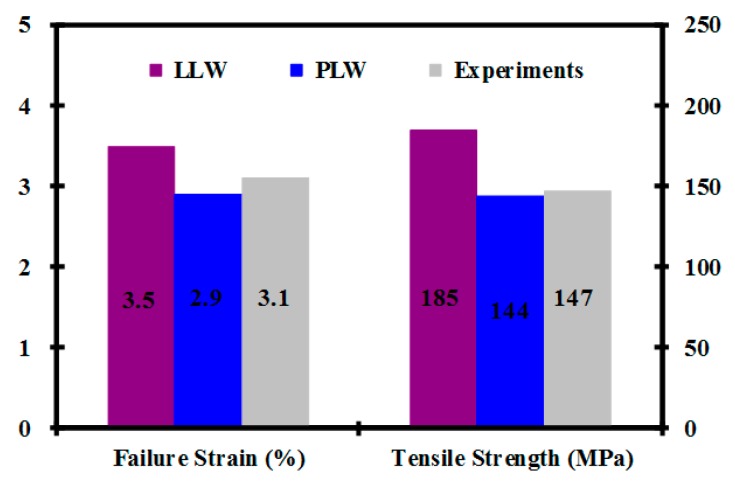
Composite tensile properties predicted by the Weibull model.

Figure 2 reveals which of the Weibull distributions can yield reasonable predictions for failure strain and tensile strength of the composite. Compared to the experimental data, the better theoretical values can be achieved through the power-law Weibull distribution. In other words, there are lower average failure strain and strength of the composite with fiber strength variability described with the PLW model. The phenomenon can be explained by noting that the average strength of the composite decreases with increasing the scatter of the fiber strength. All subsequent simulation tests are conduced on composites containing 180 fibers, and the results are based on 200 simulations for each case, owing to computation time constraints.

**Figure 3:**
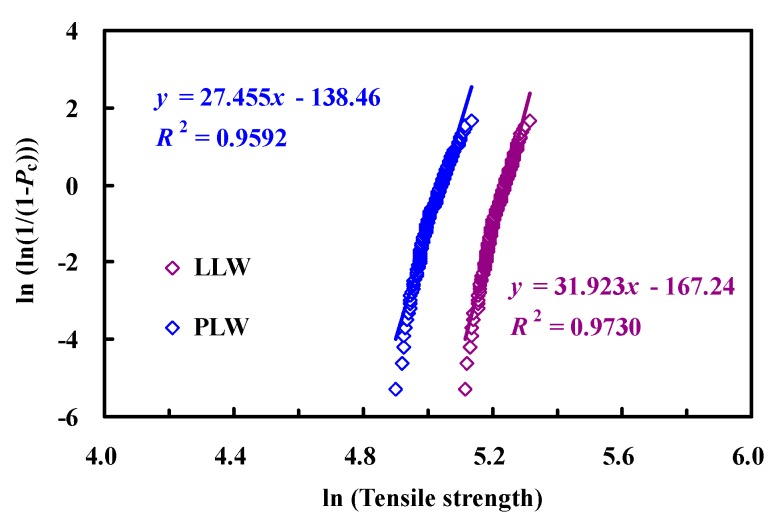
Weibull plots of the tensile strength of composites.

Figure 3 is a plot of predicted strength distribution on Weibull coordinates according to the two Weibull models. Here, *P_c_* is the cumulative failure probability estimator. From any set of 200 simulations, the 200 strengths are arranged in ascending order, $\sigma_{c}^{\left(i\right)}$, and *i* is the rank, ranging from 1 to 200. Then, the *P*_c_ associated with $\sigma_{c}^{\left(i\right)}$ is $P_{c}^{\left(i\right)}=i/\left(I+1\right)$, where *I* is the total number of simulations [20]. From a visual inspection, the data points fit best to a linear line in the case of single modal Weibull distribution [37], and this is confirmed by the relatively high *R*^2^ coefficient. It suggests that the Weibull method may be approximately applied to describe the strength variability of composites that have Weibull fiber statistics. However, it should be emphasized that the matrix and interface properties also play a significant role in modeling composite mechanical behavior [38,39].

**Figure 4:**
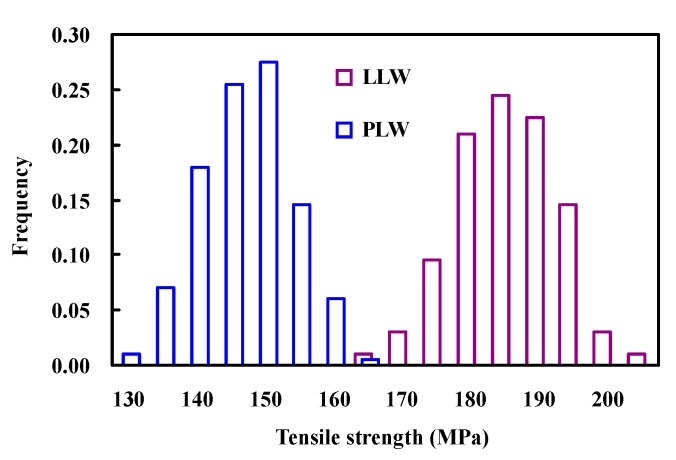
Strength distribution histogram of composites.

Figure 4 shows the histogram of the tensile strength distribution obtained from our simulation studies. In the case of PLW model, there is a weaker strength in the composite as compared to the LLW model. This is primarily due to the fact that the PLW method allows a larger variability in fiber strength, resulting in lower composite strength. As would be expected, the statistical variation of tensile strength for such composites is still affected by the fiber strength variability [36].

Table 6 presents the predictions of damage initiation and ultimate failure in the composite via the two Weibull distributions. Results of simulations using the PLW model are different from those obtained with the LLW model. Due to the presence of weak fiber elements with lower strength, the fiber damage is more likely to initiate when performing simulations with the PLW model.

**Table 6:** Predicted mechanical properties of composites using two Weibull models.

Term	Strain when the first fiber breaks (%)	Stress when the first fiber breaks (%)	Failure strain (%)	Tensile strength (MPa)
LLW	1.2	70	3.5	185
PLW	0.9	49	2.9	144

Each data is the average value of 200 simulations.

**Figure 5:**
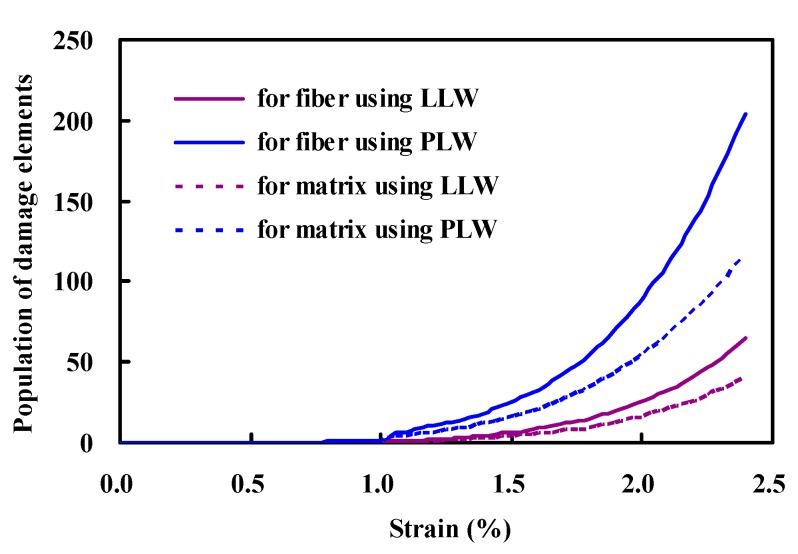
Damage evolution in the fiber and matrix during loading.

**Figure 6:**
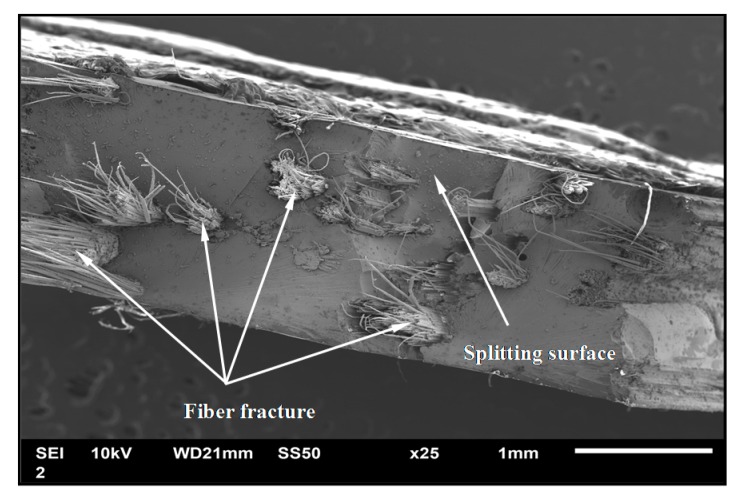
SEM image of fracture surface of composite.

Figure 5 shows the damage–strain curve for fiber and matrix, and the ordinate is represented by the average number of damage elements. There are two findings that should be addressed. One is that the PLW model leads to a higher damage growth rate, as compared with the LLW model. The difference is observed after the beginning of the initial damage in fibers, and becomes more pronounced as more fibers break. This phenomenon can be attributed to the fact that those fibers described by the PLW model exhibit larger strength variability, and thus have a lower average strength. Consequently, the stress concentrations derived from a fiber breakage may more easily cause other fibers or matrix near the failure site to fail. The second finding is that evidence of larger fiber fracture and less matrix cracking has been achieved through the simulations. Furthermore, the scanning electron micrograph that shows the fracture surface morphology of composite specimen is presented in Figure 6. It is concluded from these results that fiber fracture is prominent in the microfailure mechanism associated with the tensile behavior of such composites.

## 5. Conclusions

In this work, we have presented a multiscale approach for modeling damage evolution and predicting tensile strength in unidirectional composites containing bamboo fibers. Fiber strength variability is found to have a statistical effect on the mechanical properties of the composite. The linear-law Weibull model combined with the Monte-Carlo method is compared in depth to the predictions of the power-law Weibull model. The comparison between predicted values and experimental data reveals that the power-law Weibull model provides a more accurate expression to describe the variability of fiber strength based on the actual flaw distributions present within the natural fibers and it may be beneficial for the explanation of the strength dispersions of plant-based polymer composites. The inadequacy of this linear-law Weibull model is suspected to be because of the existence of a nonlinear distribution of flaw density within a fiber. The results highlight the need for a higher-order Weibull expression, which can account for fiber diameter variations. Also, the validity of the MDS weak-link scaling predictions for fiber strength is confirmed by comparing with experimental data. The analysis is expected to provide an insight into the sensitivity of the predictions of the composite properties to the probabilistic variations of the reinforcements. On the other hand, more influential factors, such as the 3D stress analysis, correlations of the flaw strengths along a single fiber, and the properties of matrix and interface should be considered to conduct more exact simulation in our future work.

## Abbreviations

The following abbreviations are used in this manuscript:

LLW: Linear-law Weibull
PLW: Power-law Weibull
MLE: Maximum Likelihood Estimation
